# Maternal immune activation during pregnancy is associated with more difficulties in socio-adaptive behaviors in autism spectrum disorder

**DOI:** 10.1038/s41598-023-45060-z

**Published:** 2023-10-17

**Authors:** Pierre Ellul, Anna Maruani, Valérie Vantalon, Elise Humeau, Anouck Amestoy, Andrea Anchordoqui, Paola Atzori, Jean-Marc Baleyte, Safiyah Benmansour, Olivier Bonnot, Manuel Bouvard, Ariane Cartigny, Nathalie Coulon, Romain Coutelle, David Da Fonseca, Caroline Demily, Marion Givaudan, Fanny Gollier-Briant, Fabian Guénolé, Andrea Koch, Marion Leboyer, Aline Lefebvre, Florian Lejuste, Charlotte Levy, Eugénie Mendes, Natalia Robert, Carmen M. Schroder, Mario Speranza, Elodie Zante, Hugo Peyre, Michelle Rosenzwajg, David Klatzmann, Nicolas Tchitchek, Richard Delorme

**Affiliations:** 1 Child and Adolescent Psychiatry Department, Robert Debré Hospital, University of Paris Cité, Paris, France; 2 Immunology, Immunopathology, Immunotherapy, INSERM U959, Pitié Salpétrière Hospital, Sorbonne University, Paris, France; 3 Fondation FondaMental, Créteil, France; 4 Centre Hospitalier Charles-Perrens, Pôle Universitaire de Psychiatrie de L’enfant Et de L’adolescent, Bordeaux Cedex, France; 5 Aquitaine Institute for Cognitive and Integrative Neuroscience, UMR 5287, CNRS, INCIA, University of Bordeaux, Bordeaux, France; 6 CHU de Caen, Department of Child and Adolescent Psychiatry, Caen Normandy University, Caen, France; 7 Centre Expert TSA-SDI/Centre Référent de Réhabilitation Psychosociale, CH Alpes Isère, Grenoble, France; 8 Centre d’excellence I-Mind, Centre de Référence Maladies Rares Génopsy, Pôle ADIS, Centre Hospitalier Le Vinatier, Bordeaux, France; 9 Child and Adolescent Psychiatry Unit, Salvator University Hospital, Public Assistance-Marseille Hospitals, Aix-Marseille University, Marseille, France; 10 Psychiatrie de L’enfant et de l’Adolescent, CHU and Universite de Nantes, Nantes, France; 11 INSERM, IMRB, Translational Neuropsychiatry, AP-HP, DMU IMPACT, FHU ADAPT, Univ Paris Est Créteil, 94010 Créteil, France; 12 Expert Centre for Autism and NDD, Fondation FondaMental, Department for Child and Adolescent Psychiatry, Strasbourg University Hospitals and University of Strasbourg, Versailles, France; 13 CNRS UPR 3212Service Universitaire de Psychiatrie de L’enfant Et de L’adolescent, Centre Hospitalier de VersaillesUMR1018, CESPUVSQ, Université Paris Saclay, Versailles, France; 14 Human Genetics and Cognitive Functions Unit, Pasteur Insitute, Paris, France; 15 Institute of Neuroscience Timone, CNRS, Aix-Marseille University, Marseille, France

**Keywords:** Biomarkers, Neurodevelopmental disorders

## Abstract

Autism spectrum disorder (ASD) are neurodevelopmental conditions characterised by deficits in social communication and interaction and repetitive behaviours. Maternal immune activation (MIA) during the mid-pregnancy is a known risk factor for ASD. Although reported in 15% of affected individuals, little is known about the specificity of their clinical profiles. Adaptive skills represent a holistic approach to a person's competencies and reflect specifically in ASD, their strengths and difficulties. In this study, we hypothesised that ASD individual with a history of MIA (MIA^+^) could be more severely socio-adaptively impaired than those without MIA during pregnancy (MIA^-^). To answer this question, we considered two independent cohorts of individuals with ASD (PARIS study and FACE ASD) screened for pregnancy history, and used supervised and unsupervised machine learning algorithms. We included 295 mother–child dyads with 14% of them with MIA^+^. We found that ASD-MIA^+^ individuals displayed more severe maladaptive behaviors, specifically in their socialization abilities. MIA^+^ directly influenced individual's socio-adaptive skills, independent of other covariates, including ASD severity. Interestingly, MIA^+^ affect persistently the socio-adaptive behavioral trajectories of individuals with ASD. The current study has a retrospective design with possible recall bias regarding the MIA event and, even if pooled from two cohorts, has a relatively small population. In addition, we were limited by the number of covariables available potentially impacted socio-adaptive behaviors. Larger prospective study with additional dimensions related to ASD is needed to confirm our results. Specific pathophysiological pathways may explain these clinical peculiarities of ASD- MIA^+^ individuals, and may open the way to new perspectives in deciphering the phenotypic complexity of ASD and for the development of specific immunomodulatory strategies.

## Introduction

Autism spectrum disorder (ASD) are heterogeneous neurodevelopmental conditions characterised by social communication impairment and interaction and repetitive or stereotyped behaviors. Its incidence is 1 in 50 to 1 in 100 births, affecting 52 million people worldwide^1^. The etiopathogenesis of ASD results from the close intertwining of genetic predispositions and environmental risk factors. Among known environmental insults, maternal immune activation in mid-pregnancy (MIA)—due to maternal autoimmune diseases or infections—is increasingly seen as a risk factor for ASD in offspring^2^. In accordance with epidemiological evidences, well-replicated pre-clinical studies show that this association leads to ASD by direct action of maternal immune mediators (*i.e.* cytokines) on fetal cortical neurons, disrupting normal neurodevelopment and leading to autism-like behaviours in the pups^3^. Years of intensive research have made it possible to divide the common and heterogeneous autism spectrum into rarer but etiopathogenically homogeneous entities, based mainly on common genetic variants^4^. This approach allows to identify and better understand common pathophysiological pathways and thus the emergence of potential targeted therapies. Nonetheless, few studies have attempted to characterise ASD subgroup according to common pathogenic environmental factors. Despite a history of MIA being frequent, approximatively reported in 15% of ASD individuals, there is surprisingly little data on their specific clinical profiles^5,6^.

Clinical studies often use symptom scales to assess the clinical characteristics of individuals and the severity of the condition, which do not reflect their adaptability to the environment. According to the *American Association on Intellectual and Developmental Disorders* (AAIDD), adaptive behaviour is «the collection of conceptual, social, and practical skills that all people learn in order to function in their daily lives»^7^. Adaptive skills thus represent a higher concept of functioning, taking into account all dimensions of a person in his/her environment^8^.

We hypothesized that individual with ASD and a history of MIA (MIA^+^) would display more severe socio-maladaptive behaviors than those without MIA during pregnancy (MIA^-^). To perform this study, we considered two independent cohorts of individuals with ASD (PARIS study and FACE ASD) screened for pregnancy history and used supervised and unsupervised machine learning algorithms to decipher the possible interactions between MIA and adaptive behaviors in ASD.

## Methods

### Participants recruitments

We included in our study children with ASD enrolled in the PARIS study, conducted by the Excellence Centre for Autism & Neuro-developmental Disorders (InovAND—Robert Debré Hospital, Paris, France) between March 2017 and April 2021. This study was approved by the local ethics committee (2021–27 N° IDRCB: 2021-A00489-32). Prior to inclusion, informed consent was obtained from a parent and/or legal guardian. We also enrolled ASD individuals from an independent sample from eight Expert Centers for ASD (Créteil, Bordeaux, Grenoble, Versailles, Marseille, Caen, Strasbourg, Lyon) coordinated by Fondation FondaMental. The relevant Ethical Review Board (CPP- Est IV) approved the appraisal protocol on 18 June 2019. All participants gave their informed consent. All methods in both studies were performed in accordance with relevant guidelines/regulations and have been performed in accordance with the Declaration of Helsinki. Patients with a diagnosis of MIA and information about the history of MIA were included, no family refused to participate. Considering the frequency of the MIA event (12–15%) and the number of individual per sample, we pooled all individuals to increase the power analysis of the study. Separate demographic information of each cohort is in Supplementary Table 1.

### Clinical evaluation and questionnaires

The diagnosis of ASD was performed according to DSM-5 criteria^9^ by summing up the information from the Autism Diagnostic Interview-Revised (ADI-R)^10^, the Autism Diagnostic Observation Schedule -2nd edition (ADOS-2)^11^ and clinical records of individuals. The ADI-R is a parental interview for the diagnosis of autism, which can be carried out from the age of 24 months. It reviews the child's developmental history, investigating communication, social development and play, restricted interests and behaviours, general behaviours and the age of onset of difficulties. In the ADOS-2, the child is asked to perform activities to assess communication, reciprocal social interaction, play and/or imaginative use of materials, stereotyped behaviour, restricted interests and other abnormal behaviour. Different modules are offered depending on the age and level of functioning of the subject, ranging from pre-school children to verbal adults.

All participants were screened with a parental semi-structured interview for pre- and peri- natal history exploring pregnancy complications (Threatened preterm delivery, High blood pressure, Placenta Praevia, Premature rupture of membranes, Materno-foetal infection). This interview was conducted by a child and adolescent psychiatrist specialising in ASD at best in the presence of both parents, but often only the mother. We focused on any history of MIA during pregnancy based solely on the parents' declaration. Based on this information collected by the parents, children were then split either into MIA (MIA^+^) or in non-MIA (MIA^-^) sub-groups. We considered mothers with a significant history of an MIA-related event when they were: (i) with an autoimmune disease as listed by the American Autoimmune Related Diseases Association^12^ and (ii) with a viral or bacterial infection during pregnancy with a fever over 38.5 °C for more than 24 h. Mothers with an infection resulting from a pathogen with a well-documented direct brain cytopathic effect (such as cytomegalovirus infection) were excluded. Details of MIA are in Supplementary Table 2.

The level of autonomy and adaptation of children with neurodevelopmental conditions is assessed with the second edition of the Vineland Adaptive Behavior Scale (VABS) ^8^^,^^13^. The VABS is the reference test for assessing the level of autonomy and adaptation at all ages. The Vineland-II explores 3 major areas: Communication, Daily Life Skills and Socialisation. Assessing the overall level of autonomy and ability to adapt helps in diagnosis and provides information for setting up educational and remedial programmes. The Vineland scale comprises 117 questions and takes the form of a semi-structured interview with the parents or primary carer. It can be administered and scored by a psychologist or another professional trained in the evaluation and interpretation of tests. The test takes between 30 min and 1 h to complete, depending on the person concerned. At the end of the test, for each domain assessed, the result expresses the average level of competence achieved by the person in that domain, in age equivalence. It should be note that in this study, IQ and scales for others co-occuring conditions (ADHD, etc.) were not available. Missing data for each group are in Supplementary Table 3.

### Statistical analysis

Prior to each analysis, we assessed the normality of variable distributions using the Shapiro–Wilk test. For continuous data, depending on its normality, we employed either the Student’s t-tests or the Wilcoxon tests. For categorical data, we utilized the Fisher's test. Additionally, only those linear models were adopted that demonstrated a normal distribution of residuals. We conducted hierarchical regression analyses to determine whether MIA significantly accounts for the variance in the dependent variables.. Hierarchical regression is a stepwise method where predictors are added or removed in stages or blocks. This approach facilitates the analysis of how multiple predictor variables explain variance in a dependent variable in the context of datasets which include confounding variables. Detailed results of the hierarchical regression analyses can be found in the supplementary materials. The final models from the multivariate analysis, available in the supplementary materials, were all adjusted based on sex and ASD scales (SRS T-score/ADOS total score). To infer the presence or absence of MIA based on multiple collected input variables, we used classification decision trees, which are supervised predictive machine learning models. Classification decision trees offer a visual and intuitive approach to data analysis, allowing for straightforward interpretation of complex datasets by segmenting the data into branches based on hierarchical decision rules, and facilitating the handling of both categorical and continuous variables without requiring extensive data preprocessing or assumptions about data distributions. Classification trees were generated using R package 'Party'^14^. The relevance of the groups identified by the classification trees was then tested using ANOVA with post-hoc Tukey for multiple mean comparisons. To complement the results of the classification tree and study which variables are the most important for predicting MIA, we performed supervised classification methods using the R package 'mlr3'^15^. For classification, we used both logistic regression classification learner and a linear discriminant analysis classification learner. Logistic regression classification learner predicts the probability of an outcome based on one or more predictor variables by modeling the data on a logistic function, while linear discriminant analysis classification learner seeks to find a linear combination of features that best separates two or more classes. The advantage of these methods is their ability to model relationships between predictors and categorical outcomes effectively, while providing clear coefficients or weightings for each predictor, aiding interpretability and decision-making. We divided samples into two groups: 80% for training and the remaining 20% for validation. As the MIA^+^ individuals were under-represented, we used an oversampling method with a 3.5 ratio. The sample characteristics before and after oversampling are in Supplementary Fig. 1. The learning parameters used by the model were then evaluated using the package R package 'iml'^16^ with three components: feature effects, Shapley values and feature importance. Finaly, to comprehensively understand both the direct and indirect effects of all variables on the MIA outcome across various causal pathways, we employed unsupervised path analysis utilizing the 'lavaan' R package^17^. We did not apply multiple test corrections for *p*-values since many of our models, especially Linear Discriminant Analysis (LDA) and classification decision tree, did not produce *p*-value outputs. Statistical analysis was performed using R studio version 4.2.1.

### Ethical approval

As part of the PARIS study, this study was approved by the local ethics committee of Robert Debré Hospital (2021–27 N° IDRCB: 2021-A00489-32). Informed consents were obtained from patients or a parent and/or legal guardian before enrollment in the study. All methods in both studies was performed in accordance with relevant guidelines/regulations and have been performed in accordance with the Declaration of Helsinki.

## Results

### General characteristics

295 mother–child dyads were included in our study, with a history of MIA reported in 14% of mothers (n = 40) (MIA^+^). Details of MIA is on Supplementary Table 2. Dyads without a history of MIA (n = 255, 86%) were used as a comparison group (MIA^-^).

Demographic information, prenatal history, birth parameters and ASD scales are provided in Table 1.

**Table 1:** Demographic, prenatal; birth parameters and autistic severity profile of the population studies.

	MIA −	MIA +	Overall	Test statistic
	(n = 255)	(n = 40)	(n = 295)	
Age (month) mean (SD)	109.57 (49.42)	100.17 (40.66)	108.30 (48.37)	Wilcoxon rank-sum *p*-value = 0.195
Gender N (Col %)				Fisher exact *p*-value 0.356
Female	43 (16.93%)	4 (10.26%)	16.04%	
Male	211 (83.07%)	35 (89.74%)	83.96%	
Pre-natal history				
Threatened preterm delivery N (Col %)				Fisher exact *p*-value = 0.304
No	240 (94.12%)	39 (90.00%)	93.56%	
Yes	15 (5.88%)	4 (10.00%)	6.44%	
High blood pressure N (Col %)				Fisher exact *p*-value = 0.630
No	247 (96.86%)	38 (95.00%)	96.61%	
Yes	8 (3.14%)	2 (5.00%)	3.39%	
Placenta Praevia N (Col %)				Fisher exact *p*-value = 0.090
No	253 (99.22%)	38 (95.00%)	98.64%	
Yes	3 (0.78%)	2 (5.00%)	1.36%	
Premature rupture of membranes N (Col %)				Fisher exact *p*-value = 0.002
No	255 (100.00%)	37 (92.50%)	98.98%	
Yes	0 (0.00%)	3 (7.50%)	1.02%	
Materno-foetal infection N (Col %)				Fisher exact *p*-value = 0.019
No	253 (99.22%)	37 (92.50%)	98.31%	
Yes	2 (0.78%)	3 (7.50%)	1.69%	
Birth parameters				
Weight (g) Median (IQR)	3370.00 (715.00)	3420.00 (545.00)	3372.50 (698.75)	Wilcoxon rank-sum *p*-value = 0.964
Height (cm) Median (IQR)	50.00 (3.00)	50.00 (4.00)	50.00 (3.00)	Wilcoxon rank-sum *p*-value = 0.257
Head Circumference (cm) Median (IQR)	35.00 (1.50)	35.00 (3.00)	35.00 (2.00)	Wilcoxon rank-sum *p*-value = 0.2
APGAR 1 min Median (IQR)	10.00 (0.00)	10.00 (1.00)	10.00 (0.00)	Wilcoxon rank-sum *p*-value = 0.053
APGAR 5 min Median (IQR)	10.00 (0.00)	10.00 (0.00)	10.00 (0.00)	Wilcoxon rank-sum *p*-value = 0.088
ASD scales				
ADOS (total score) Median (IQR)	18.00 (8.00)	16.00 (9.75)	18.00 (9.00)	Wilcoxon rank-sum *p*-value = 0.487
SRS (T-score) Median (IQR)	73.00 (16.00)	76.00 (16.50)	73.00 (16.00)	Wilcoxon rank-sum *p*-value = 0.668
VABS – Communication subdomain score Median (IQR)	67.00 (36.00)	64.00 (38.00)	67.00 (34.00)	Wilcoxon rank-sum *p*-value = 0.308
VABS—Daily Living Skills subdomain score Median (IQR)	69.00 (24.50)	66.00 (19.25)	68.50 (23.50)	Wilcoxon rank-sum *p*-value = 0.441
VABS—Socialisation subdomain score Median (IQR)	64.00 (34.50)	57.00 (36.25)	63.00 (33.50)	Wilcoxon rank-sum *p*-value = 0.239

MIA: Maternal immune activation, SRS: Social Responsiveness Scale 2nd edition; ADOS Autism Diagnostic Observation Schedule 2nd edition; VABS: Vineland adaptative behavior scale second edition.

We observed that mothers with MIA during pregnancy had more history of premature rupture of membranes (PROM) (*p*-value = 0.002) and maternofetal infection (MFI) (*p*-value = 0.02) than those without MIA. ASD-MIA^+^ individuals did not differ from those without a history of MIA in terms of symptom severity estimated with the SRS T-score (*p*-value = 0.6) or ADOS total score (*p*-value = 0.4). Univariate analysis did not reveal any difference in any of the three sub-domain of the VABS: communication (*p*-value = 0.3), socialisation (*p*-value = 0.2), daily living skills (DLS) (*p*-value = 0.4) domains.

### More severe impact on adaptative behaviors related to socialization is associated with MIA during pregnancy

**Figure 1:**
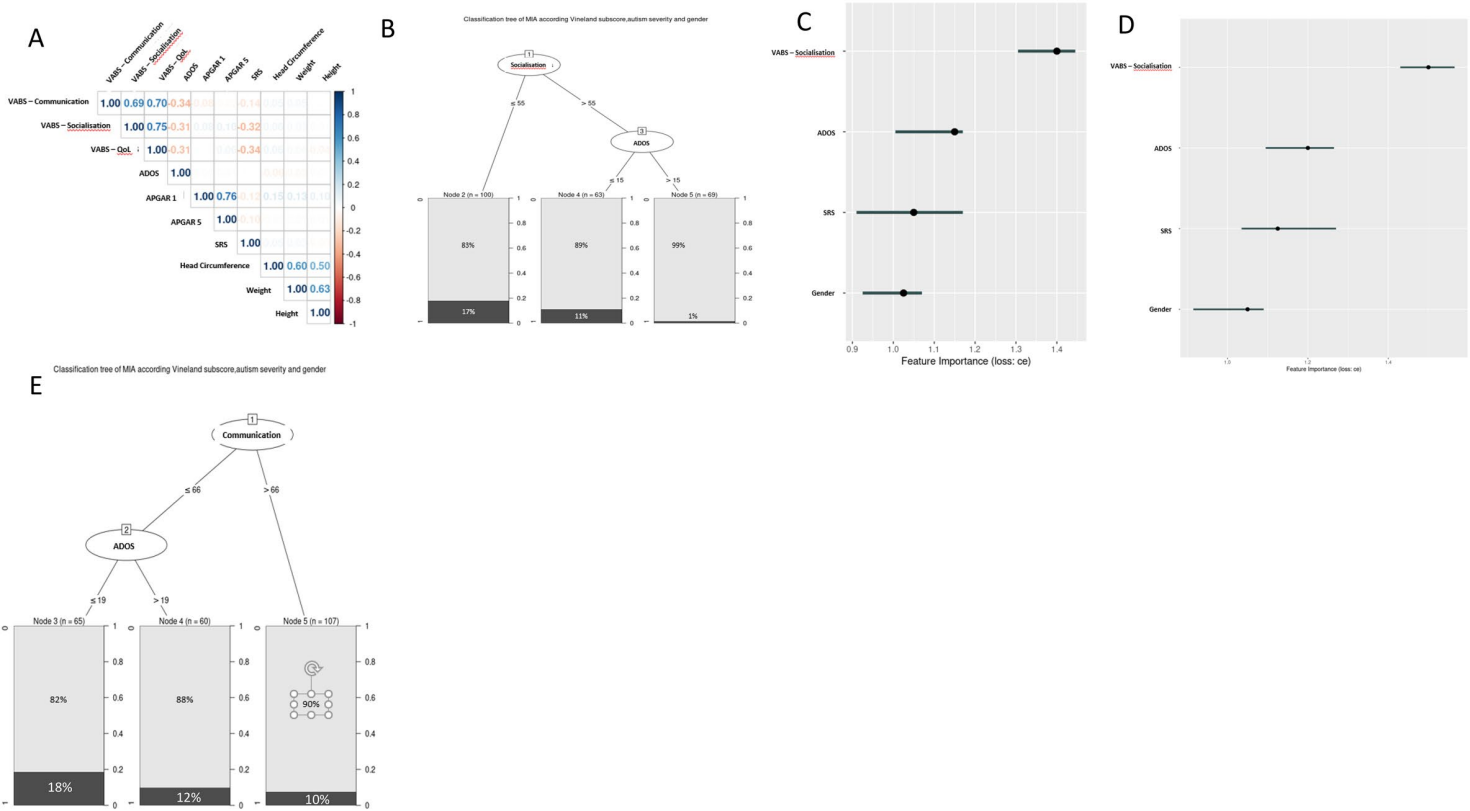
More severe impact on adaptative behaviors related to socialization is associated with maternal immune activation during pregnancy. (**A**) Correlogram of the different subdomain of the Vineland II with clinical parameters. (**B**) Classification decision tree of the risk of maternal immune activation during pregnancy depending on Vineland II—Socialisation sub-score and ADOS total score on the autistic offspring. (**C**) Feature importance for the logistic analysis. Feature importance computes the importance of features by calculating the increase in the model prediction error after permuting the feature. (**D**) Feature importance for the linear discriminant analysis. (**E**) Classification decision tree of the risk of maternal immune activation during pregnancy depending on Vineland II—Communication sub-score and ADOS total score on the autistic offspring. MIA: Maternal immune activation, SRS: Social Responsiveness Scale 2nd edition; ADOS Autism Diagnostic Observation Schedule 2nd edition, VABS: Vineland Adaptive Behavior Scales 2nd edition.

We found, that SRS T-score and ADOS total score were correlated with the three sub-scores of VABS (Fig. 1A). Accordingly, hierarchical regression analysis showed that more severe socio-maladaptive behaviors in ASD individuals were associated with a MIA during pregnancy (*p*-value = 0.006) (Supplementary Table 4 and Table 5). To confirm this association, we used a classification tree which isolated three groups (Fig. 1B): (i) a MIA low-probability group (VABS—Socialisation > 55 and ADOS total score > 15) with 1% of MIA^+^; (ii) a medium-probability group (VABS—Socialisation > 55 and ADOS total score =  < 15) with 11% of MIA^+^; and (iii) a high-probability group (VABS—Socialisation =  < 55) with 17% of MIA^+^. Significant differences between the high-probability group and the low-probability group were observed (*p*-value = 0.002).

Finally, we validated these results using two distinct supervised classification algorithms. Using logistic classification, we discriminated MIA^+^ individuals with an accuracy of 70%, a sensitivity of 84%, a specificity of 27% and an area under the curve (AUC) of 72%. In accordance with our initial hypothesis, the most robust parameter to classify children was the VABS—Socialisation score followed by ADOS total score (Fig. 1C and Supplementary Fig. 2A and 2B). Using linear discriminant analysis classification (LDA), we reported similar results with an accuracy of 72%, a sensitivity of 84%, a specificity of 33% and AUC of 66%. Of note, the VABS—Socialisation score was also the most important parameter allowing classification, but in LDA, both ADOS total score and SRS T-score were pertinent to classify children (Fig. 1D). Considering the communication domain, we observed that a lower score was also associated with a history of MIA (*p*-value = 0.045) (Supplementary Table 6). We next used a classification tree also isolating three groups of individuals (Fig. 1E): (i) a MIA low-probability group (VABS—communication > 66) with 10% of MIA^+^ ; (ii) a medium-probability group (VABS—Communication =  < 66 and ADOS total score > 19) with 12% of MIA^+^ ; and (iii) a high-probability group (VABS—Communication =  < 66 and ADOS total score < 19)) with 18% of MIA^+^. No significant differences were found between groups (*p*-value < 0.08). Thus, we considered that the communication domain of the VABS was not associated with MIA. Lastly, no association was observed between DLS and MIA (*p*-value = 0.2) (Supplementary Table 7).

### MIA may directly affect the socio-maladaptive behaviors in ASD

We observed that MIA was associated with more pregnancy complications also known to increase independently the risk for ASD (Table 1)^18^. Pregnancy complications and more severe ASD symptomatology were correlated with more adaptive behaviours later on (Supplementary Fig. 3). Adjusting for these potential factors and corroborating our previous results, we investigated whether a history of MIA was associated with poorer adaptive behavior and if so, the mediator of this association.

**Figure 2:**
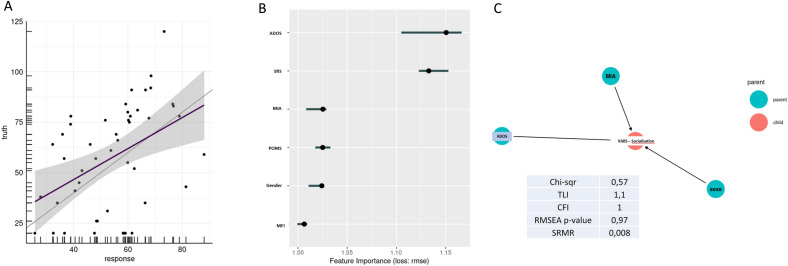
Maternal immune activation may directly affected the socio-maladaptive behaviors in autism children. (**A**) Predictions of the linear model on socialization sub scores compared to measured scores. (**B**) Feature Iimportance of the linear regression model. (**C**) Path analysis on the influence of MIA on socialization sub-score. MIA: Maternal immune activation, SRS: Social Responsiveness Scale 2nd edition; ADOS Autism Diagnostic Observation Schedule 2nd edition, VABS: Vineland Adaptive Behavior Scales 2nd edition, PROM: Premature rupture of membranes; MFI: Materno-fetal infection; TLI: Tucker-Lewis index; CFI comparative fit index; RMSEA root mean square error of approximation; SRMR: standardized root mean squared residual.

Using adjustment models, we found no association between the history of MIA and communication (*p*-value = 0.15) or DLS (*p*-value = 0.63) sub-domain but a significant association with poorer socio-adaptive behaviours (*p*-value = 0.03) (Supplementary Table 8, 9 and 10). Analysis with a linear model regression learner confirmed this association (mean absolute error = 20.2; mean square error = 562.7; root mean square error = 23.7, R-square = 0.16) (Fig. 2A).

According to feature importance, MIA appeared to predict the intensity of communication deficit, just after the intensity of the global autistic symptom severity itself (Fig. 2B and Supplementary Fig. 4). Finally, we explored whether there was a causal link between the MIA and the impairment intensity of socio-adaptive skills. We used structural equation modelling and applied a path analysis to our dataset. We found, with a good model performance (Fig. 2C), that MIA directly influenced the socio-adaptive skills of children, independently of pregnancy complications or global severity of ASD symptoms (Fig. 2C).

## Discussion

We found that ASD individuals with a history of MIA during pregnancy displayed more difficulties in socio-adaptive skills. For individuals with a VABS socialization subdomain score < 55, almost 20% of children had a history of MIA, compared to 1–10% for those with a VABS socialization subdomain score > 55. Social-adaptive skills are a central node of ASD symptomatology. Compared to healthy children, ASD children, no matter the MIA status, have more difficulties in adaptive social behaviors with other adaptive domains less impacted^19^. Our results were consistent with the literature with exploratory study reporting more severe symptoms of social impairment—assessed with the SRS- in case of immune activation during pregnancy (in case of asthma and allergies)^5^. One limitation is that we did not assess all the clinical variables likely to influence socio-adaptive behaviour. Intelligence quotient, for example, was not integrated, whereas there were significant entanglements between IQ and adaptive functioning^20^. This limitation must be contrasted with a previous study finding that MIA was not associated withdecreased cognitive functions^6^. Future studies should integrate all the dimensions of ASD children and frequent comorbidity, such as ID or ADHD, in order to decipher the socio-adaptive abnormalities in ASD children^21^.

We also observed that MIA directly influenced the socio-adaptive behaviors of ASD individuals The effect of MIA did not seem to bemediated by an increase in the severity of autistic symptoms. This hypothesis mirrored findings in children with neurodevelopmental conditions (excluding ASD), in whom a history of MIA was associated, in a dose–response effect, with more externalizing and internalizing problems^22^. In mice, MIA directly triggers autistic-like symptoms through an effect on neuronal cytokine receptors in the cerebral cortex^3^. Immunogenetic factors may also mediate the link between MIA, mother and unborn child. Indeed, a variant in HLA-G has been found in ASD patients but also in their mothers^23^. HLA-G is mainly expressed in placental tissues playing a role in maternal–fetal tolerance^24^ and could, therefore, modulate the impact of MIA on neurodevelopment. MIA also appears to disrupt neurodevelopment by altering microglia function, leading to deficits in synaptic pruning and abnormalities in synaptic connectivity^25–27^. However, MIA also induced epigenetic changes of major transcriptional factors in the offspring that are independent of the onset of ASD core symptoms^28^. This effect is mediated by the maternal microbiota and leads to a long-term immune imbalance in the offspring, characterised by a peripheral increase in the Th17 lymphocyte subtype (Th17)^29^. Th17 are major pro-inflammatory lymphocytes and are in a constant and dynamic equilibrium with its anti-inflammatory counterpart regulatory T lymphocytes (Tregs)^30,31^. Interestingly, ASD individuals displayed a peripheral increase in Th17 and a decrease in Tregs, which was more pronounced in those with a history of MIA^32^. Interestingly, a recent study on mice demonstrated that specific stimulation of Tregs in ASD pups from MIA mothers reverses autistic-like symptoms^33^. Overall, this suggests that the epigenetically mediated peripheral immune imbalance induced by MIA may participate in the socio-maladaptive behaviors in ASD and could be targeted by specific immunomodulatory strategies.

## Limitations

One of the main limitations of our study is intrinsically linked to its retrospective design, which leads to a possible recall bias concerning MIA during pregnancy and, in particular, the timing of MIA in our cohort. To overcome this problem, we used a strict definition of MIA, but this will not replace a prospective study to confirm our results. Even if we pooled two cohorts, the population remains relatively small and to better decipher the influence of MIA, many clinical and biological factors need to be studied on larger cohorts to obtain the necessary statistical power. Nevertheless, all our results were validated by different and complementary statistical approaches with good model performance, highlighting the robustness of our data.

Finally, one additional limitation of our study was the limited number of covariables which potentially impacted socio-adaptive behaviors, that we included in the analysis. Intelligence quotient, for example, was not integrated, whereas there were significant entanglements between IQ and adaptive functioning^20^. In addition, we were unable to assess other dimensions of ASD, such as repetitive/restricted behaviors. Future studies should integrate additional dimensions related to ASD, such as intellectual developmental disorder or Attention Deficit / Hyperactive Disorder, to further decipher the impact of MIA on socio-adaptive impairment in ASD^21^.

## Conclusions

MIA may affect in the long term the socio-adaptive behavioral trajectories of individuals with ASD. Specific pathophysiological pathways may explain these clinical peculiarities of ASD- MIA^+^ individuals, and may open the way to new perspectives in deciphering the phenotypic complexity of ASD and the development of targeted immunotherapy strategies.

### Supplementary Information

Supplementary Figure 1.

Supplementary Figure 2.

Supplementary Figure 3.

Supplementary Figure 4.

Supplementary Table 1.

Supplementary Table 2.

Supplementary Table 3.

Supplementary Tables.

## Data Availability

The PARIS dataset and the code used during the current study are available from the corresponding author upon request. Due to ethical and legal restrictions, data involving clinical participants of the FACE-ASD cohort cannot be made publicly available. All relevant data are available upon request to the Fondation FondaMental for researchers who meet the criteria for access to confidential data.

## Abbreviations

ADOS: Autism Diagnostic Observation Schedule 2nd edition
ASD: Autism spectrum disorders
MIA: Maternal immune activation
SRS: Social Responsiveness Scale 2nd edition
VABS: Vineland Adaptive Behavior Scales 2nd edition
