# Supplementary material for: Maternal immune activation during pregnancy is associated with more difficulties in socio-adaptive behaviors in autism spectrum disorder
Source: Sci Rep. 2023 Oct 17;13:17687. doi: 10.1038/s41598-023-45060-z (PMC10582088; doi:10.1038/s41598-023-45060-z)
Supplement: Supplementary file 1 — Supplementary Figure 1. [file 41598_2023_45060_MOESM1_ESM.pptx]

## Slide 1
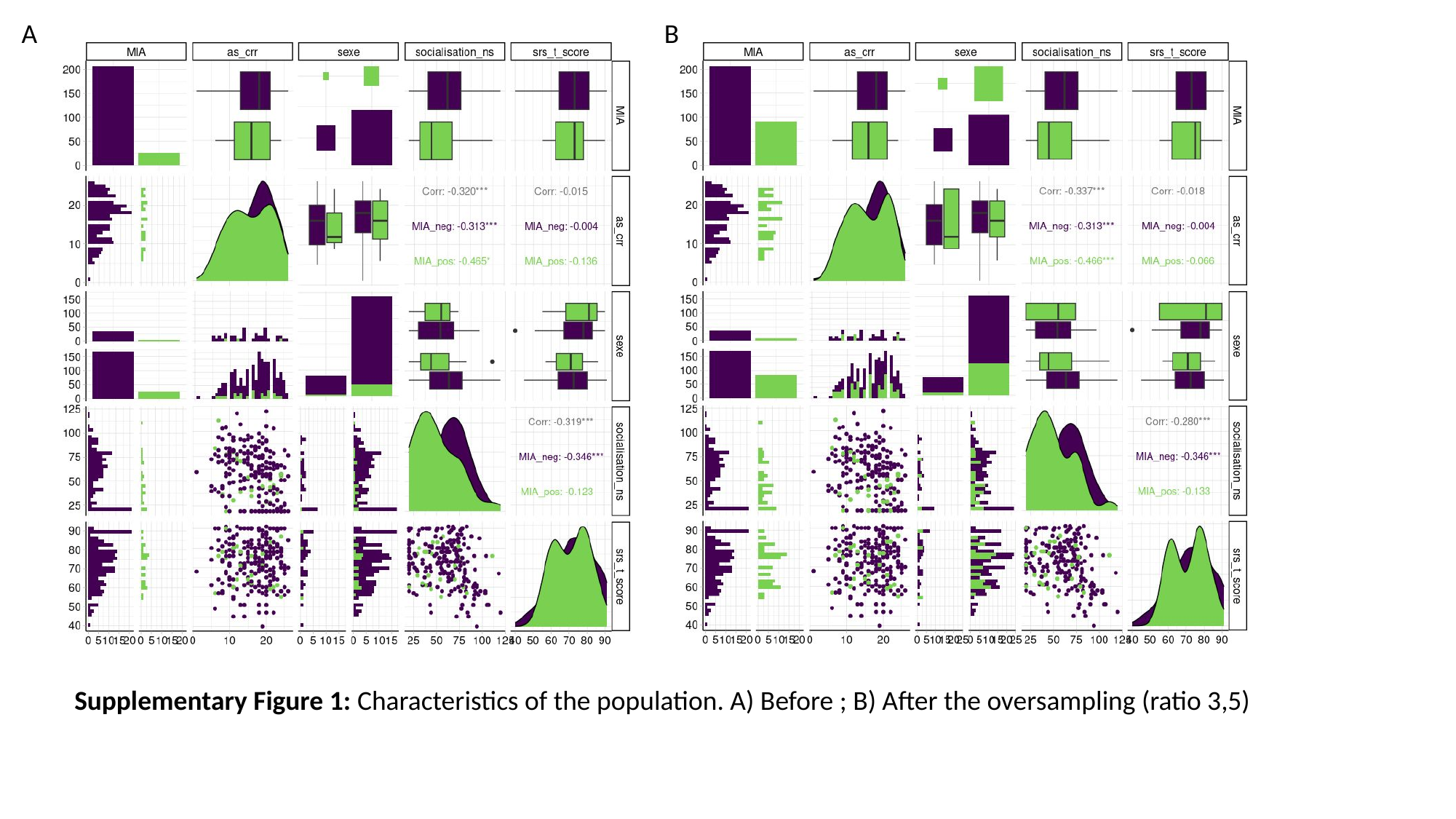

A
B
Supplementary Figure 1: Characteristics of the population. A) Before ; B) After the oversampling (ratio 3,5)
