# Supplementary material for: Maternal immune activation during pregnancy is associated with more difficulties in socio-adaptive behaviors in autism spectrum disorder
Source: Sci Rep. 2023 Oct 17;13:17687. doi: 10.1038/s41598-023-45060-z (PMC10582088; doi:10.1038/s41598-023-45060-z)
Supplement: Supplementary file 3 — Supplementary Figure 3. [file 41598_2023_45060_MOESM3_ESM.pptx]

## Slide 1
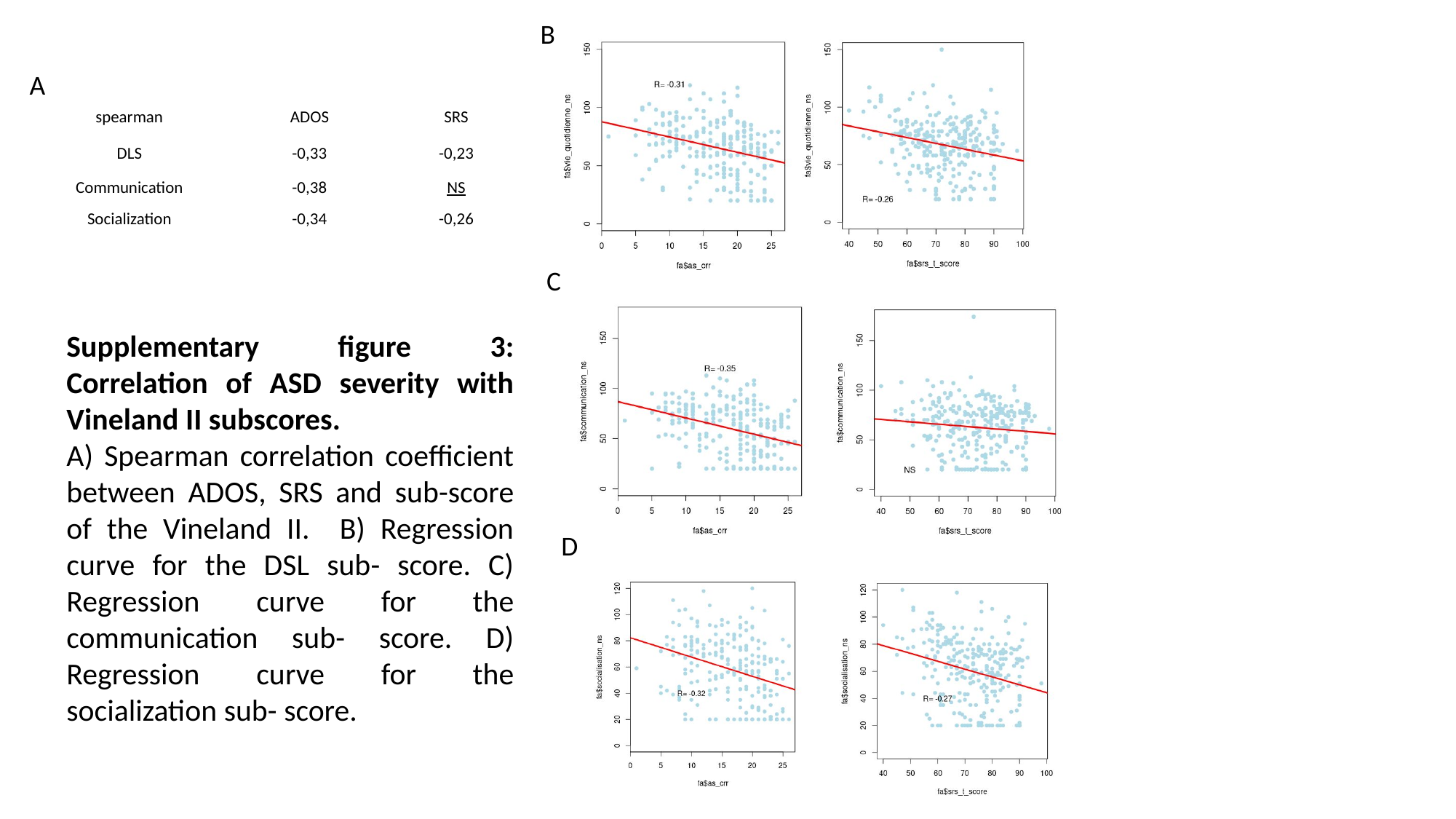

B
A
| spearman | ADOS | SRS |
| --- | --- | --- |
| DLS | -0,33 | -0,23 |
| Communication | -0,38 | NS |
| Socialization | -0,34 | -0,26 |
C
Supplementary figure 3: Correlation of ASD severity with Vineland II subscores.
A) Spearman correlation coefficient between ADOS, SRS and sub-score of the Vineland II. B) Regression curve for the DSL sub- score. C) Regression curve for the communication sub- score. D) Regression curve for the socialization sub- score.
D
