# Supplementary material for: Maternal immune activation during pregnancy is associated with more difficulties in socio-adaptive behaviors in autism spectrum disorder
Source: Sci Rep. 2023 Oct 17;13:17687. doi: 10.1038/s41598-023-45060-z (PMC10582088; doi:10.1038/s41598-023-45060-z)
Supplement: Supplementary file 4 — Supplementary Figure 4. [file 41598_2023_45060_MOESM4_ESM.pptx]

## Slide 1
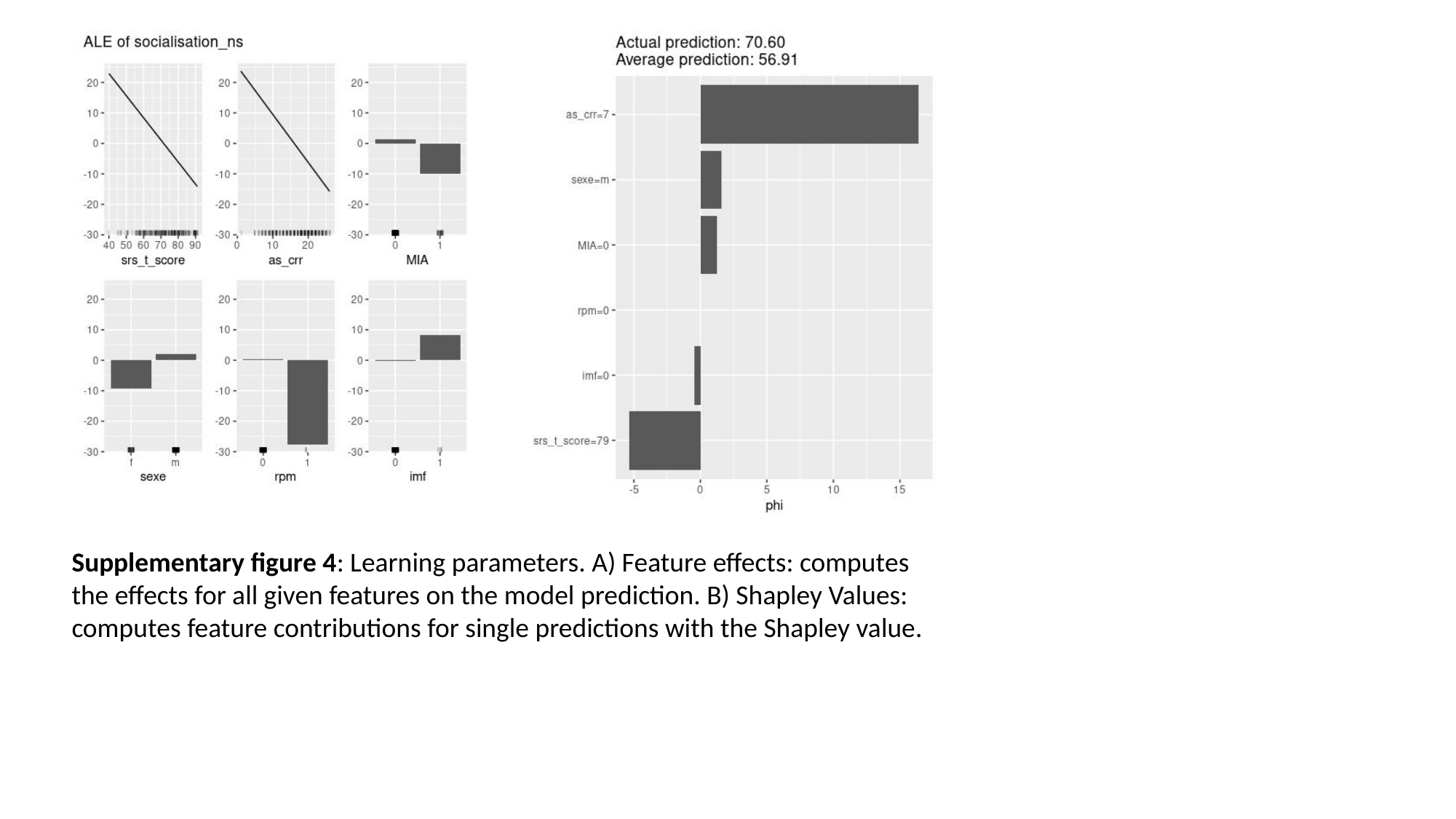

Supplementary figure 4: Learning parameters. A) Feature effects: computes the effects for all given features on the model prediction. B) Shapley Values: computes feature contributions for single predictions with the Shapley value.
