# Supplementary material for: Maternal immune activation during pregnancy is associated with more difficulties in socio-adaptive behaviors in autism spectrum disorder
Source: Sci Rep. 2023 Oct 17;13:17687. doi: 10.1038/s41598-023-45060-z (PMC10582088; doi:10.1038/s41598-023-45060-z)
Supplement: Supplementary file 5 — Supplementary Table 1. [file 41598_2023_45060_MOESM5_ESM.docx]

|  | **Expert Centers Cohort**  **N= 86** | **Paris Cohort**  **N= 209** | **Overall**  **N= 295** | **Test Statistic** |
| --- | --- | --- | --- | --- |
| **Age (month)**  **mean (SD)** | 150.14 (65.38) | 91.08 (23.22) | 108.30 (48.37) | Wilcoxon rank-sum  p-value <0.001 |
| **Gender**  N (Col %) |  |  |  | Fisher exact  p-value= 0.117 |
| Female | 9 (10.59%) | 38 (18.27%) | 16.04% |  |
| Male | 76 (89.41%) | 170 (81.73%) | 83.96% |  |
| **Maternal Immune Activation**  N (Col %) |  |  |  | Fisher exact  p-value= 0.454 |
| No | 72 (83.72%) | 183 (87.56%) | 86.44% |  |
| Yes | 14 (16.28%) | 26 (12.44%) | 13.56% |  |

Supplementary table 1 : Demographic data of the two cohorts used
