# Supplementary material for: Maternal immune activation during pregnancy is associated with more difficulties in socio-adaptive behaviors in autism spectrum disorder
Source: Sci Rep. 2023 Oct 17;13:17687. doi: 10.1038/s41598-023-45060-z (PMC10582088; doi:10.1038/s41598-023-45060-z)
Supplement: Supplementary file 6 — Supplementary Table 2. [file 41598_2023_45060_MOESM6_ESM.docx]

| **Auto-immune diseases** | **n= 23** |
| --- | --- |
| Coeliaque disease | 2 |
| Systemic Lupus erythematosus | 1 |
| Antiphospholipide syndrome | 1 |
| Type 1 Diabetes | 4 |
| Insulin-dependent gestational diabetes | 9 |
| Hashimoto thyroiditis | 6 |
| **Infections** | **n= 17** |
| Parasistis infection | 3 |
| Viral infection | 4 |
| Bacterial infection | 11 |

Supplementary table 2 : Details of maternal immune activation
