# Supplementary material for: Maternal immune activation during pregnancy is associated with more difficulties in socio-adaptive behaviors in autism spectrum disorder
Source: Sci Rep. 2023 Oct 17;13:17687. doi: 10.1038/s41598-023-45060-z (PMC10582088; doi:10.1038/s41598-023-45060-z)
Supplement: Supplementary file 7 — Supplementary Table 3. [file 41598_2023_45060_MOESM7_ESM.docx]

| Missing data |  | **MIA - = 255** | **MIA + = 40** |
| --- | --- | --- | --- |
| **Prenatal** |  |  |  |
|  | **Threatened preterm delivery (TPD)** | 0 | 0 |
|  | **High blood pressure (HBP)** | 0 | 0 |
|  | **Placenta Praevia** | 0 | 0 |
|  | **Premature rupture of membranes (PROM)** | 0 | 0 |
|  | **Materno-foetal infection (MFI)** | 0 | 0 |
|  | **Gender** | 1 (0,4) | 1 (2,5) |
| **Birth parameters** |  |  |  |
|  | **Height(cm)** | 5 (1,9) | 1 (2,5) |
|  | **Weight (g)** | 4 (1,5) | 1 (2,5) |
|  | **Head Circumference (cm)** | 9 (3,5) | 1(2,5) |
|  | **APGAR 1 minute** | 8 (3,1) | 1 (2,5) |
|  | **APGAR 5 minutes** | 8 (3,1) | 1 (2,5) |
| **Autistic scales** | **SRS** | 10 (3,9) | 1 (2,5) |
|  | **ADOS** | 44 (17) | 14 (35) |
|  | **Vineland - Communication** | 2 (0,8) | 0 (0) |
|  | **Vineland - Socialisation** | 4 (1,5) | 0 (0) |
|  | **Vineland - DLS** | 3(1,2) | 0 (0) |

Supplementary table 3 : Missing data
