# Supplementary material for: Maternal immune activation during pregnancy is associated with more difficulties in socio-adaptive behaviors in autism spectrum disorder
Source: Sci Rep. 2023 Oct 17;13:17687. doi: 10.1038/s41598-023-45060-z (PMC10582088; doi:10.1038/s41598-023-45060-z)
Supplement: Supplementary file 8 — Supplementary Tables. [file 41598_2023_45060_MOESM8_ESM.docx]

| **MIA** | **Adjustment** | **Standardized coefficient (Beta)** | **p-value** | **R-squared** | **F-statistics** |
| --- | --- | --- | --- | --- | --- |
|  | **SRS** | -0.01 | 0,77 | 0,0003 | 0,08 |
|  |  |  |  |  |  |
|  | **ADOS** | -0.05 | 0.41 | 0.002 | 0.65 |
|  |  |  |  |  |  |
|  | **Gender** | 0.06 | 0.29 | 0.003 | 1.11 |

**Supplementary table 4**: Hierarchical logistic regression analysis of maternal immune activation risk

| **MIA** | **Adjustment parameters** | **Standardized coefficient (Beta)** | **p-value** | **Adjusted R-squared** | **F-statistics** | **F-Statistics**  **p-value** |
| --- | --- | --- | --- | --- | --- | --- |
|  |  |  |  |  |  |  |
|  | **Socialization** | -0.06 | 0.25 | 0.001 | 1.30 | 0.25 |
|  |  |  |  |  |  |  |
|  | **Socialization** | -0.07 | 0.19 | 0.003 | 1.45 | 0.23 |
|  | **Gender** | 0.06 | 0.23 |  |  |  |
|  |  |  |  |  |  |  |
|  | **Socialization** | -0.19 | 0.008** | 0.02 | 2.63 | 0.05 |
|  | **ADOS** | -0.11 | 0.09 . |  |  |  |
|  | **SRS** | -0.09 | 0.17 |  |  |  |
|  |  |  |  |  |  |  |
|  | **Socialization** | -0.20 | **0.006**** | 0.02 | 2.30 | 0.05 |
|  | **ADOS** | -0.12 | 0.06 . |  |  |  |
|  | **SRS** | -0.09 | 0.1 |  |  |  |
|  | **Gender** | 0.07 | 0.2 |  |  |  |

**Supplementary table 5**: Hierarchical logistic regression results of Maternal Immune activation with socialization domain of the Vineland II

| **MIA** | **Adjustment parameters** | **Standardized coefficient (Beta)** | **p-value** | **Adjusted R-squared** | **F-statistics** | **F-Statistics**  **p-value** |
| --- | --- | --- | --- | --- | --- | --- |
|  | Communication | -0.06229872 | 0.288 | 0.0004 | 1.134 | 0.28 |
|  |  |  |  |  |  |  |
|  | Communication | -0.06502999 | 0.279 | -0.002 | 0.6341 | 0.53 |
|  | SRS | -0.02536795 | 0.673 |  |  |  |
|  |  |  |  |  |  |  |
|  | Communication | -0.13139116 | 0.0592 . | 0.009 | 2.128 | 0.12 |
|  | ADOS | -0.09942199 | 0.1528 |  |  |  |
|  |  |  |  |  |  |  |
|  | Communication | -0.06807536 | 0.247 | 0.001 | 1.25 | 0.28 |
|  | Gender | 0.06384414 | 0.277 |  |  |  |
|  |  |  |  |  |  |  |
|  | Communication | -0.13606285 | 0.0552 . | 0.007 | 1.55 | 0.20 |
|  | ADOS | -0.10330756 | 0.1407 |  |  |  |
|  | SRS | -0.05193216 | 0.4332 |  |  |  |
|  |  |  |  |  |  |  |
|  | **Communication** | -0.1433161 | **0.045 *** | 0.007 | 1.439 | 0.22 |
|  | ADOS | -0.1136296 | 0.110 |  |  |  |
|  | SRS | -0.0473376 | 0.477 |  |  |  |
|  | Gender | 0.0709228 | 0.287 |  |  |  |

**Supplementary table 6**: Hierarchical logistic regression results of Maternal Immune activation with communication domain of the Vineland II

| **MIA** | **Adjustment parameters** | **Standardized coefficient (Beta)** | **p-value** | **Adjusted R-squared** | **F-statistics** | **F-Statistics**  **p-value** |
| --- | --- | --- | --- | --- | --- | --- |
|  | **Daily living skills** | -0.0278677 | 0.635 | -0.002 | 0.2254 | 0.63 |
|  |  |  |  |  |  |  |
|  | **Daily living skills** | -0.03828426 | 0.537 | -0.005 | 0.2211 | 0.80 |
|  | **SRS** | -0.02465316 | 0.691 |  |  |  |
|  |  |  |  |  |  |  |
|  | **Daily living skills** | -0.06706829 | 0.331 | -0.01 | 0.773 | 0.46 |
|  | **ADOS** | -0.07174592 | 0.299 |  |  |  |
|  |  |  |  |  |  |  |
|  | **Daily living skills** | -0.02695630 | 0.648 | -0.002 | 0.632 | 0.53 |
|  | **Gender** | 0.06084012 | 0.303 |  |  |  |
|  |  |  |  |  |  |  |
|  | **Daily living skills** | -0.08510099 | 0.248 | -0.003 | 0.7308 | 0.53 |
|  | **ADOS** | -0.08045248 | 0,251 |  |  |  |
|  | **SRS** | -0.05761443 | 0.410 |  |  |  |
|  |  |  |  |  |  |  |
|  | **Daily living skills** | -0.08843395 | 0.234 | -0.004 | 0.7442 | 0.56 |
|  | **ADOS** | -0.08994131 | 0.207 |  |  |  |
|  | **SRS** | -0.05425238 | 0.440 |  |  |  |
|  | **Gender** | 0.06300676 | 0.348 |  |  |  |

**Supplementary table 7**: Hierarchical logistic regression results of Maternal Immune activation with daily living skills domain of the Vineland II

| **Communication** | **Adjustment parameters** | **Standardized coefficient (Beta)** | **p-value** | **AdjustedR-squared** | **F-statistics** | **F-Statistics**  **p-value** |
| --- | --- | --- | --- | --- | --- | --- |
|  | **MIA** | -0.06229872 | 0.288 | 0.0004 | 1 134 | 0.28 |
|  |  |  |  |  |  |  |
|  | **SRS** | -0.1101958 | 0.0641. | 0.008 | 3 454 | 0.06 |
|  |  |  |  |  |  |  |
|  | **ADOS** | -0.3554406 | 1.82e-08 *** | 0.12 | 33.98 | 1.825e-08 |
|  |  |  |  |  |  |  |
|  | **Gender** | 0.01216747 | 0.836 | -0.003 | 0.04279 | 0.83 |
|  |  |  |  |  |  |  |
|  | **PROM** | -0.149 | 0.0106 * | 0.018 | 6 625 | 0.01 |
|  |  |  |  |  |  |  |
|  | **MFI** | -0.0096474 | 0.869 | -0.003 | 0.027 | 0.86 |
|  |  |  |  |  |  |  |
|  | **MIA** | -0.06426162 | 0.2793 | 0.009 | 2 316 | 0.10 |
|  | **SRS** | -0.11136553 | 0.0613. |  |  |  |
|  |  |  |  |  |  |  |
|  | **MIA** | -0.1151114 | 0.0592. | 0.13 | 18.98 | 2.306e-08 |
|  | **ADOS** | -0.3615093 | 9.54e-09 *** |  |  |  |
|  |  |  |  |  |  |  |
|  | **MIA** | -0.06833665 | 0.247 | -0.002 | 0.6944 | 0.50 |
|  | **Gender** | 0.01647376 | 0.780 |  |  |  |
|  |  |  |  |  |  |  |
|  | **MIA** | -0.0258 | 0.6674 | 0.01 | 3 396 | 0.03 |
|  | **PROM** | -0.142 | 0.0182 * |  |  |  |
|  |  |  |  |  |  |  |
|  | **MIA** | -0.0625 | 0.294 | -0.002 | 0.5653 | 0.56 |
|  | **MFI** | 0.00148 | 0.980 |  |  |  |
|  |  |  |  |  |  |  |
|  | **MIA** | -0.1167668 | 0.0552. | 0.14 | 14.48 | 1.138e-08 |
|  | **ADOS** | -0.1535045 | 0.0119 * |  |  |  |
|  | **SRS** | -0.3592825 | 1.1e-08 *** |  |  |  |
|  |  |  |  |  |  |  |
|  | **MIA** | -0.02984395 | 0.6226 | 0.013 | 2 357 | 0.07 |
|  | **PROM** | -0.149718 | 0.0156 * |  |  |  |
|  | **MFI** | 0.0328 | 0.5873 |  |  |  |
|  |  |  |  |  |  |  |
|  | **MIA** | -0.092134 | 0.1561 | 0.16 | 8 379 | 3.26e-08 |
|  | **ADOS** | -0.3563 | 1.96e-08 *** |  |  |  |
|  | **SRS** | -0.15254 | 0.0125 * |  |  |  |
|  | **Gender** | 0.10346 | 0.0910. |  |  |  |
|  | **PROM** | -0.123387 | 0.0607. |  |  |  |
|  | **MFI** | 0.04415 | 0.4841 |  |  |  |

**Supplementary table 8**: Hierarchical regression analysis and multiple linear regression results of communication domain of the Vineland II

| **DLS** | **Adjustment parameters** | **Standardized coefficient (Beta)** | **p-value** | **Adjusted R-squared** | **F-statistics** | **F-Statistics**  **p-value** |
| --- | --- | --- | --- | --- | --- | --- |
|  | **MIA** | -0.0278677 | 0.635 | -0.002 | 0.2254 | 0.63 |
|  |  |  |  |  |  |  |
|  | **SRS** | -0.2600021 | 9.73e-06 *** | 0.06 | 20.3 | 9.73e-06 |
|  |  |  |  |  |  |  |
|  | **ADOS** | -0.3173865 | 6.38e-07 *** | 0.09 | 26.21 | 6.384e-07 |
|  |  |  |  |  |  |  |
|  | **Gender** | 0.0131812 | 0.823 | -0.003 | 0.05005 | 0.82 |
|  |  |  |  |  |  |  |
|  | **PROM** | -0,12 | 0,03* | 0,01 | 4,42 | 0.03 |
|  |  |  |  |  |  |  |
|  | **MFI** | -0.02 | 0,6 | -0.002 | 0.2 | 0.61 |
|  |  |  |  |  |  |  |
|  | **MIA** | -0.03570392 | 0.537 | 0.06 | 10.32 | 4.749e-05 |
|  | **SRS** | -0.26052688 | 9.59e-06 *** |  |  |  |
|  |  |  |  |  |  |  |
|  | **MIA** | -0.06046618 | 0.331 | 0.09 | 13.58 | 2.645e-06 |
|  | **ADOS** | -0.32043758 | 5.24e-07 *** |  |  |  |
|  |  |  |  |  |  |  |
|  | **MIA** | -0.02705058 | 0.648 | -0.006 | 0.1297 | 0.87 |
|  | **Gender** | 0.01481735 | 0.802 |  |  |  |
|  |  |  |  |  |  |  |
|  | **MIA** | 0,003 | 0,95 | 0.008 | 2,2 | 0.11 |
|  | **PROM** | -0,123 | 0,04* |  |  |  |
|  |  |  |  |  |  |  |
|  | **MIA** | -0,02 | 0,69 | -0.005 | 0,2 | 0.81 |
|  | **MFI** | -0,02 | 0,66 |  |  |  |
|  |  |  |  |  |  |  |
|  | **MIA** | -0.0683941 | 0.248 | 0.19 | 19.56 | 2.532e-11 |
|  | **ADOS** | -0.3212925 | 1.33e-07 *** |  |  |  |
|  | **SRS** | -0.3233929 | 1.14e-07 *** |  |  |  |
|  |  |  |  |  |  |  |
|  | **MIA** | 0,003 | 0,95 | 0,004 | 1,46 | 0.22 |
|  | **PROM** | -0,123 | 0,04* |  |  |  |
|  | **MFI** | 0,0003 | 0,99 |  |  |  |
|  |  |  |  |  |  |  |
|  | **MIA** | -0,029 | 0.63 | 0.20 | 10,95 | 1.063e-10 |
|  | **ADOS** | -0,32 | 1.33e-07 *** |  |  |  |
|  | **SRS** | -0,32 | 1.37e-07 *** |  |  |  |
|  | **Gender** | 0,08 | 0.1520 |  |  |  |
|  | **PROM** | -0,12 | 0.0545. |  |  |  |
|  | **MFI** | -0,002 | 0.9685 |  |  |  |

**Supplementary table 9**: Hierarchical regression analysis and multiple linear regression results of DLS domain of the Vineland II

| **Socialization** | **Adjustment parameters** | **Standardized coefficient (Beta)** | **p-value** | **Adjusted R-squared** | **F-statistics** | **F-Statistics**  **p-value** |
| --- | --- | --- | --- | --- | --- | --- |
|  | **MIA** | -0.06700383 | 0.255 | 0.001 | 1 303 | 0.25 |
|  |  |  |  |  |  |  |
|  | **SRS** | -0.2751609 | 2.83e-06 *** | 0.07 | 22.85 | 2.831e-06 |
|  |  |  |  |  |  |  |
|  | **ADOS** | -0.3224902 | 3.88e-07 *** | 0.10 | 27.28 | 3.882e-07 |
|  |  |  |  |  |  |  |
|  | **Gender** | 0.07365257 | 0.212 | 0.001 | 1 565 | 0.21 |
|  |  |  |  |  |  |  |
|  | **PROM** | -0.1423187 | 0.0151 * | 0.01 | 5 975 | 0.015 |
|  |  |  |  |  |  |  |
|  | **MFI** | 0.005363 | 0.927 | -0.003 | 0.008313 | 0.92 |
|  |  |  |  |  |  |  |
|  | **MIA** | -0.07852375 | 0.173 | 0.07 | 12.4 | 6.969e-06 |
|  | **SRS** | -0.27650956 | 2.47e-06 *** |  |  |  |
|  |  |  |  |  |  |  |
|  | **MIA** | -0.1442862 | 0.0193 * | 0.11 | 16.68 | 1.694e-07 |
|  | **ADOS** | -0.3300970 | 1.72e-07 *** |  |  |  |
|  |  |  |  |  |  |  |
|  | **MIA** | -0.07719739 | 0.191 | 0.004 | 1 643 | 0.19 |
|  | **Gender** | 0.07861543 | 0.183 |  |  |  |
|  |  |  |  |  |  |  |
|  | **MIA** | -0.03275922 | 0.5874 | 0.01 | 3 128 | 0.04 |
|  | **PROM** | -0.13394328 | 0.0271 * |  |  |  |
|  |  |  |  |  |  |  |
|  | **MIA** | -0.0701 | 0.241 | -0.002 | 0.694 | 0.50 |
|  | **MFI** | 0.017826 | 0.766 |  |  |  |
|  |  |  |  |  |  |  |
|  | **MIA** | -0.1530136 | 0.00888 ** | 0.21 | 22.89 | 5.259e-13 |
|  | **ADOS** | -0.3307837 | 3.41e-08 *** |  |  |  |
|  | **SRS** | -0.3305254 | 3.60e-08 *** |  |  |  |
|  |  |  |  |  |  |  |
|  | **MIA** | -0.038629 | 0.5256 | 0.013 | 2 292 | 0.07 |
|  | **PROM** | -0.14437 | 0.0201 * |  |  |  |
|  | **MFI** | 0.04807 | 0.428 |  |  |  |
|  |  |  |  |  |  |  |
|  | **MIA** | -0.13412777 | **0.0307 *** | 0.23 | 13.07 | 1.091e-12 |
|  | **ADOS** | -0.33179819 | 3.86e-08 *** |  |  |  |
|  | **SRS** | -0.32672640 | 4.61e-08 *** |  |  |  |
|  | **Gender** | 0.12034687 | 0.0393 * |  |  |  |
|  | **PROM** | -0.12090432 | 0.0537. |  |  |  |
|  | **MFI** | 0.06603244 | 0.2724 |  |  |  |

**Supplementary table 10**: Hierarchical regression analysis and multiple linear regression results of socialization domain of the Vineland II
